# Me vs. the machine? Subjective evaluations of human- and AI-generated advice

**DOI:** 10.1038/s41598-025-86623-6

**Published:** 2025-02-01

**Authors:** Merrick R. Osborne, Erica R. Bailey

**Affiliations:** https://ror.org/01an7q238 grid.47840.3f 0000 0001 2181 7878 U.C. Berkeley, Haas School of Business, Berkeley, United States

**Keywords:** Psychology, Human behaviour

## Abstract

Artificial intelligence (“AI”) has the potential to vastly improve human decision-making. In line with this, researchers have increasingly sought to understand how people view AI, often documenting skepticism and even outright aversion to these tools. In the present research, we complement these findings by documenting the performance of LLMs in the personal advice domain. In addition, we shift the focus in a new direction—exploring how interacting with AI tools, specifically large language models, impacts the user’s view of themselves. In five preregistered experiments (*N =* 1,722), we explore evaluations of human- and ChatGPT-generated advice along three dimensions: quality, effectiveness, and authenticity. We find that ChatGPT produces superior advice relative to the average online participant even in a domain in which people strongly prefer human-generated advice (dating and relationships). We also document a bias against ChatGPT-generated advice which is present only when participants are aware the advice was generated by ChatGPT. Novel to the present investigation, we then explore how interacting with these tools impacts self-evaluations. We manipulate the order in which people interact with these tools relative to self-generation and find that generating advice before interacting with ChatGPT advice boosts the quality ratings of the ChatGPT advice. At the same time, interacting with ChatGPT-generated advice before self-generating advice decreases self-ratings of authenticity. Taken together, we document a bias towards AI in the context of personal advice. Further, we identify an important externality in the use of these tools—they can invoke social comparisons of me vs. the machine.

**Supplementary Information:**

The online version contains supplementary material available at 10.1038/s41598-025-86623-6.

## Main

Few technological innovations have integrated into modern life as rapidly as artificial intelligence (AI), in particular, the use of large language models (LLMs). These models harness vast datasets of human-generated text to deliver seemingly effortless responses across a wide range of topics. With the widespread availability of tools like ChatGPT, Claude, and Gemini, everyday users can now seek AI-generated input on a broad swath of personal and professional matters. Such interactions have been praised as a means of enhancing human decision-making^1–3^. As a result, LLMs have become increasingly woven into the fabric of daily life, from powering web browsers^4^to assisting with homework^5^. More and more, people are turning to these tools to guide their choices and behaviors.

However, there remains a domain where humans may still hold a distinct edge over AI: providing personal advice. When seeking guidance on matters such as interpersonal relationships, personal development, or health, individuals may prefer to seek human input, as they may believe that other humans offer unique insights and lived experiences that can produce superior advice. Thus, for personal advice, the value of human experience can amplify the tendency to shy away from algorithmic suggestions—a phenomenon known as “algorithm aversion”^6^. This aversion manifests when people favor human-generated content over AI output^7^, even when AI demonstrates superior performance.

In the present research, we investigate whether LLMs, specifically ChatGPT, can produce high-quality, effective, and authentic personal advice. We also explore how algorithm aversion plays out in personal advice contexts, examining whether individuals change their perceptions of AI-generated advice once they are aware it comes from an LLM. Moreover, we analyze how interacting with AI tools like ChatGPT affects users’ perceptions of their own self-generated advice.

Our findings offer three key contributions. First, we extend the algorithm aversion literature by identifying appraisal factors—quality, effectiveness, and authenticity—that lead to decreased uptake of advice, despite high evaluations when the LLM source is undisclosed. Second, we contribute to the evolving body of research on LLMs by examining how engagement with AI-generated advice influences self-perception, a novel addition to our understanding of the implications of these tools. Finally, we consider the sequence in which LLMs are integrated into human tasks, particularly in advice generation scenarios, exploring how starting with either AI-generated or self-generated content shapes participants’ evaluations.

## Theoretical background

Research on algorithm aversion highlights a human tendency to distrust algorithmic decisions or recommendations, even when these outperform human-generated alternatives^6,8^. Numerous researchers have considered the conditions under which this aversion occurs^9,10^. On the other hand, the phenomenon of “algorithmic appreciation” describes instances where individuals recognize and embrace the strengths of algorithmic solutions, such as their ability to process large datasets quickly and accurately or maintain objectivity in decision-making, suggesting that the type of task is a key moderator in the presence of algorithmic aversion^11,12^.

In the present research, we extend robust research on algorithm aversion by focusing on a novel domain: personal advice. Unlike tasks such as stock price prediction^13^or song ranking^14^, personal advice is inherently less formulaic with a non-numeric output. Yet, there are reasons to believe AI, specifically large language models (LLMs), can offer high-quality advice. Emerging research has documented the utility of AI-generated advice in medical^15^and mental health domains^16,17^. In addition, being trained on text data and communicating in a conversational, human-like fashion gives LLMs a unique ability to mimic human-generated content in a way that may be surprisingly useful in the advice domain.

At the same time, this conversational capacity may have implications for self-perceptions by triggering social comparison processes. Novel to the present investigation, we suggest that using LLMs can invoke the psychological process of social comparison. Social comparison has been a robust topic of research since it was introduced by Leon Festinger in the 1950s^18–20^. Social comparison is a nearly automatic form of self-evaluation relative to a referent person or social group^21–23^. In the present research, we focus on self-evaluation, which is related to assessments of one’s ability to generate valuable content (e.g., “How good is my advice?”). Although past research on social comparison has largely examined human-to-human comparisons, we propose that human-to-machine comparisons will emerge during interactions with LLMs—and may even amplify self-assessment disparities.

When people engage in “me-to-machine” comparisons, their self-evaluations may be impacted as they are likely to construct an evaluation of themselves *relative *to the human-like but distinctly nonhuman agent. Given that LLMs synthesize a vast range of human-generated perspectives, their recommendations may resemble “the wisdom of crowds”^24,25^ rather than a single person’s viewpoint. This dynamic can produce mixed outcomes: on one hand, users may feel validated if they perceive themselves as “better than average”; on the other, they may experience a sense of alienation or inadequacy, as they compare themselves to what seems like error-reduced, crowd-informed expertise. Indeed, me-to-machine comparisons represent a particularly unique—and potentially challenging—form of social comparison, as the benchmark for comparison is nonhuman.

Our empirical investigation comprised five preregistered studies (*N =* 1,195) evaluating LLM performance in the personal advice domain, documenting biases against GPT-generated advice, and exploring the self-evaluative impacts of engaging with these tools. In Study 1, we explored the general preference for human- vs. AI-generated advice across a set of personal advice topics (e.g., wellness, finance, and relationships). We then explore evaluations of human- vs. AI-generated advice along three dimensions: effectiveness, quality, and authenticity (Study 2). Next, we manipulate the advice source to document a bias against AI-generated advice (Study 3). Finally, Studies 4–5 explore how exposure to ChatGPT advice impacts self-evaluations and whether these evaluations are dependent on knowingly evaluating AI-generated advice.

Taken together, our investigation seeks to unravel the underlying determinants contributing to the observed preferences and perceptions of AI-generated advice. By interrogating the interplay between AI and human-generated personal advice, this project aims to contribute to our understanding of the complext interactions between AI and us.

## Results

**Fig. 1:**
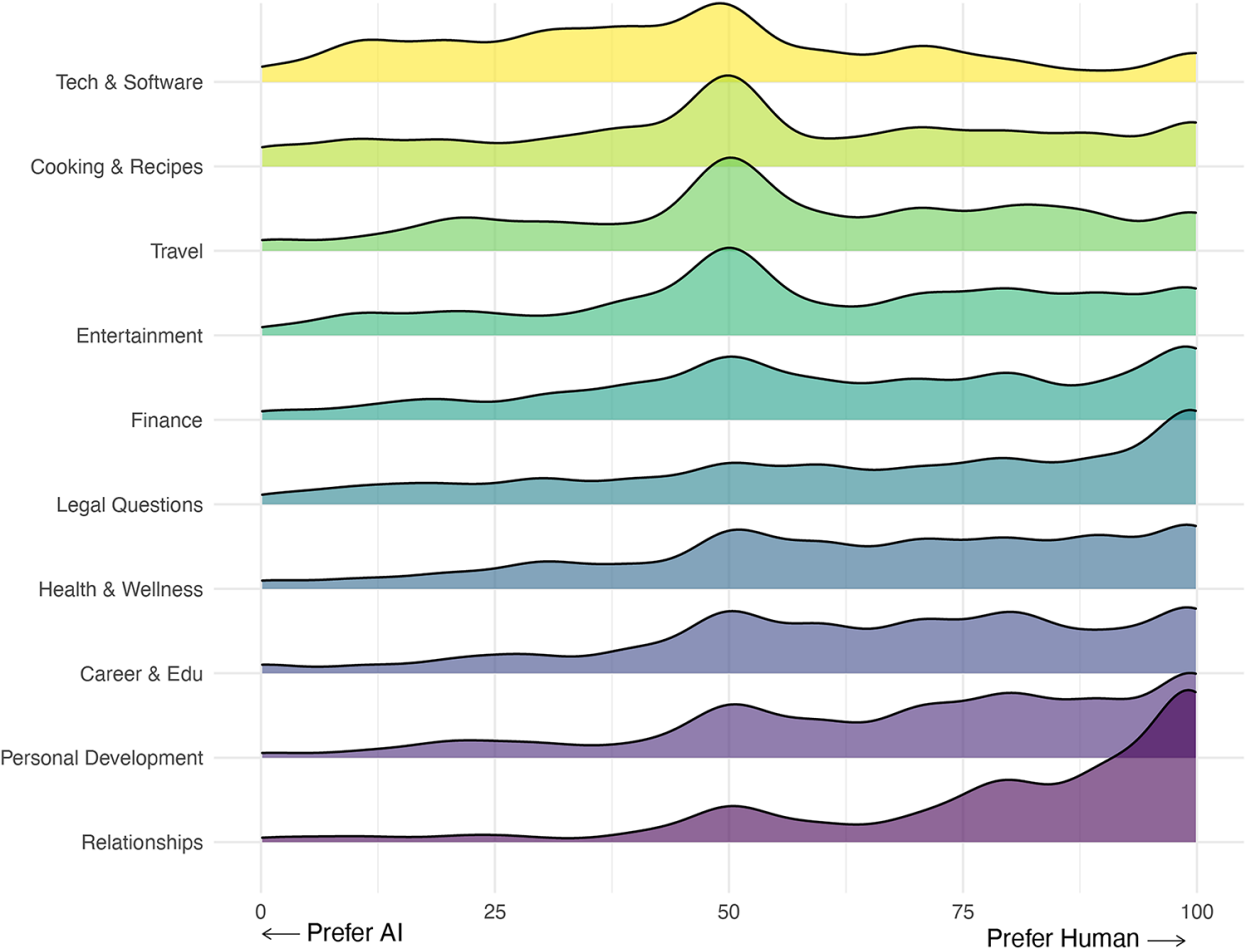
Advice Preferences for AI- versus Human-Generated Advice by Topic. Figure 1 displays a density ridge visualization of the distribution of preferences for artificial intelligence (AI) versus human-generated advice across the set of topics. Colors represent unique tasks, ordered by the strength of the preference for human-generated advice.

In Study 1, participants indicated their preferences for human- vs. AI-generated advice across a set of ten topics (e.g., finance, travel, health & wellness). We asked participants to indicate if they would prefer getting advice from an AI or a human, on a scale of 0 (preference for AI-generated advice) to 100 (preference for human-generated advice) where the midpoint of the scale (50) indicated “no preference.” Averaged across all topics, we found evidence for AI-aversion in the advice domain: on average, people significantly preferred human-generated advice (M = 62.87, SD = 28.01; mean difference = 12.87, 95% CI [60.83, 64.91], *t*(282) = 12.40, *p <* .001; Cohen’s d = 0.74). Looking across the ten topics, only one (“Technology and software”) showed a preference for AI-generated advice (mean = 46.08; see Fig. 1). The topic in which people preferred human advice *the most* (and therefore AI advice the least) was “Dating and Relationships” (see Table 1). Participants preferred advice from humans significantly more than the next-closest topic from which people wanted advice from humans: “Personal development” (*p* < .001, *d* = 0.42). Given this, we utilized the context of dating and relationships for the subsequent studies.

**Table 1:** Preferences for AI- vs. human-generated advice by topic. Table 1 presents the mean preference for human (vs. AI) generated advice in study 1 coded such that 0 = *strongly prefer AI*, 50 = *no preference*, and 100 = *strongly prefer human.* The columns represent the mean preference (“M”) and standard deviation (“SD”). The shaded portion of the table presents the results of a one-sample t-test comparing the mean to 50% (no preference) with associated *t*-statistic, *p*-value, 95% confidence interval (“Lower CI” and “Upper CI”), and Cohen’s d effect sizes.

Category	M	SD	Relative to “No Preference” (50)				
			t	*p*-value	Cohen’s d	Lower CI	Upper CI
Tech & Software	46.08	26.41	−2.50	.013	−0.15	42.99	49.17
Cooking & Recipes	54.42	28.56	2.60	.009	0.15	51.08	57.76
Travel	57.37	26.35	4.71	< .001	0.28	54.29	60.45
Entertainment	59.34	27.06	5.80	< .001	0.35	56.17	62.50
Finance	63.08	27.57	7.98	< .001	0.47	59.86	66.31
Health & Wellness	65.07	26.34	9.62	< .001	0.57	61.98	68.15
Legal Questions	65.98	29.41	9.14	< .001	0.54	62.53	69.42
Career & Education	66.15	25.35	10.72	< .001	0.64	63.19	69.12
Personal Development	70.62	25.14	13.80	< .001	0.82	67.68	73.56
Relationships	80.61	23.20	22.20	< .001	1.32	77.90	83.33

**Fig. 2:**
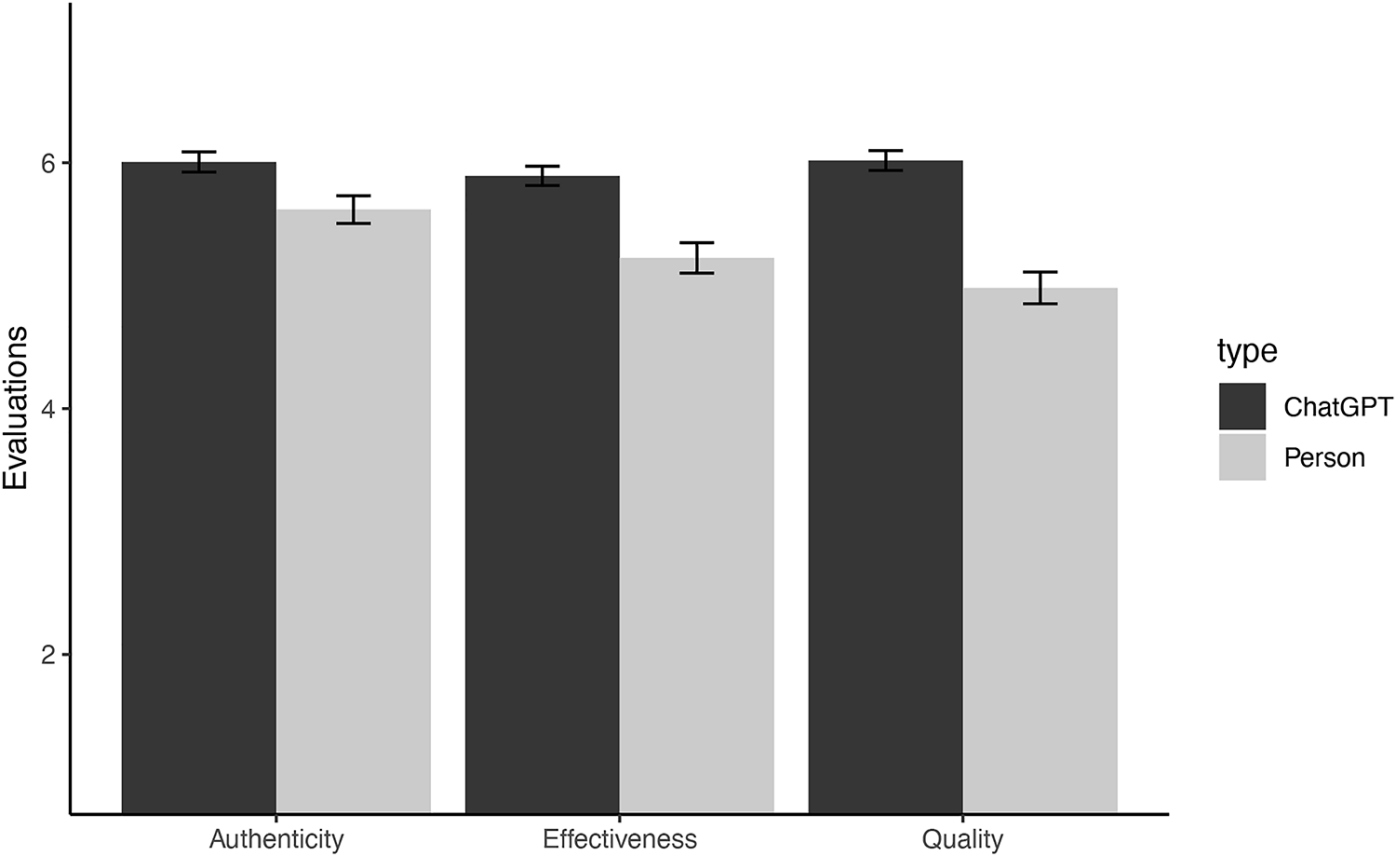
Advice Evaluations for ChatGPT- and Human-Generated Advice. The figure displays the evaluation of the advice by participants. The evaluations of ChatGPT are presented in the darker bars, with evaluations of human-generated advice in the lighter bars. The bars represent the mean, and the error bars represent the standard errors.

In Study 2, we explored subjective evaluations of human- vs. AI-generated advice in the context of dating and relationships in a two-stage study. The goal of this study was to establish a “neutral” evaluation of ChatGPT- and human-generated advice in the context of dating and relationships, a context where people were particularly averse to AI-generated advice. To test this, we first asked a group of MTurkers (*N =* 95), as well as ChatGPT (*N =* 1), for advice regarding a specific dating scenario. We then fed responses to a second group of MTurk participants (*N =*168) who were asked to evaluate the effectiveness, quality, and authenticity of two pieces of advice, the ChatGPT-generated advice and one randomly selected piece of advice from the first MTurk sample. Participants in Study 2 were not provided with the source of either piece of advice. In line with research highlighting how ChatGPT can outperform humans^25^(or, at least, present an argument as compellingly as a person^26^), participants evaluated the ChatGPT advice as more effective, higher quality, and more authentic relative to the human-generated advice (see Table 2; Fig. 2). We replicated this effect in Supplemental Studies A & B with two additional LLMs, Claude by Anthropic and Gemini by Google, finding the same pattern of results (see Supplemental Information for more details).

**Table 2:** Within-participant comparisons for advice evaluations (study 2). Table 2 presents the mean evaluations for human (vs. AI) generated advice in study 2. The columns represent the mean evaluations for ChatGPT and human-generated advice (“M”) and their corresponding standard deviations (“SD”). The right-hand side shaded portion of the table presents the results of within-participant *t-*tests with the mean difference between the variables, with associated *t*-statistic, *p-*value, 95% confidence interval (“Lower CI” and “Upper CI”), and Cohen’s d effect sizes.

Variable	TARGET				Mean diff.	t	*p*-value	Cohen’s d	Lower CI	Upper CI
	ChatGPT		Human							
	M	SD	M	SD						
Effectiveness	5.89	1.02	5.23	1.61	0.67	4.83	< .001	0.37	0.39	0.94
Quality	6.02	1.05	4.98	1.67	1.04	7.06	< .001	0.54	0.75	1.33
Authenticity	6.01	1.07	5.62	1.46	0.39	2.82	.005	0.22	0.12	0.66

In Study 3, we documented the bias against AI-generated advice by presenting participants with the GPT-generated advice from Study 2. We randomized what participants were told the source of the advice was; in the “overt ChatGPT condition,” participants were told that the advice was generated by ChatGPT. In the “covert ChatGPT condition,” participants were told that the advice was generated by another online worker. Participants in both conditions were presented with the same advice generated by ChatGPT. We found that participants evaluated the ChatGPT advice as significantly less effective, lower quality, and less authentic relative to the “human-generated” version of the same advice (see Table 3). We also explored whether there were differences in the recommendation of that advice by condition (“advice uptake”). We found that participants recommend lower advice uptake in the overt ChatGPT condition relative to the covert ChatGPT condition (see Table 3).

**Table 3:** Between-participant comparisons for advice evaluations (Study 3). Table 3 presents the mean evaluations for overt ChatGPT-generated advice relative to Covert ChatGPT-generated advice in study 3. In both conditions, the advice was the same. We manipulated the source of the advice to participants. The columns represent the mean evaluations for our four dependent variables (“M”) and their corresponding standard deviations (“SD”). The right-hand side shaded portion of the table presents the results of between-subjects *t-*tests with the mean difference between the variables, with associated *t*-statistic, *p-*value, 95% confidence interval (“Lower CI” and “Upper CI”), and Cohen’s d effect sizes.

Variable	CONDITION				Mean diff.	t	*p*-value	Cohen’s d	Lower CI	Upper CI
	Overt ChatGPT		Covert ChatGPT							
	M	SD	M	SD						
Effectiveness	5.30	1.34	5.85	1.09	−0.55	5.21	.001	−0.45	0.34	0.76
Quality	5.41	1.19	5.74	1.04	−0.34	3.47	< .001	−0.30	0.15	0.53
Authenticity	5.02	1.46	5.78	1.10	−0.76	6.82	< .001	−0.59	0.54	0.99
Advice Uptake	5.32	1.29	5.72	1.14	−0.40	3.81	< .001	−0.33	0.20	0.61

In Studies 4–5, we then moved to explore the social comparison aspect of advice generation and evaluation by asking participants to generate their own advice in coordination with reading and evaluating ChatGPT-generated advice. This was done in a counterbalanced order with some participants generating and evaluating their advice before reading and evaluating ChatGPT-generated advice, or vice versa. This order makes salient the me-to-machine effect: in the case of self-generation first, the participant has their own advice to compare the ChatGPT advice against. In the case of GPT evaluation first, the participant has the GPT advice to compare their own against. Thus, the salient me-to-machine comparison is observed in the GPT-first condition, where participants are evaluating their advice with the knowledge of the GPT-generated advice.

In Study 4, we found that participants evaluated the AI-generated advice as similar in terms of quality and effectiveness to their advice (Cohen’s d’s < 0.09). However, they rated their own advice as significantly more authentic relative to AI-generated advice (Cohen’s d = 0.72; see Table 4).

**Fig. 3:**
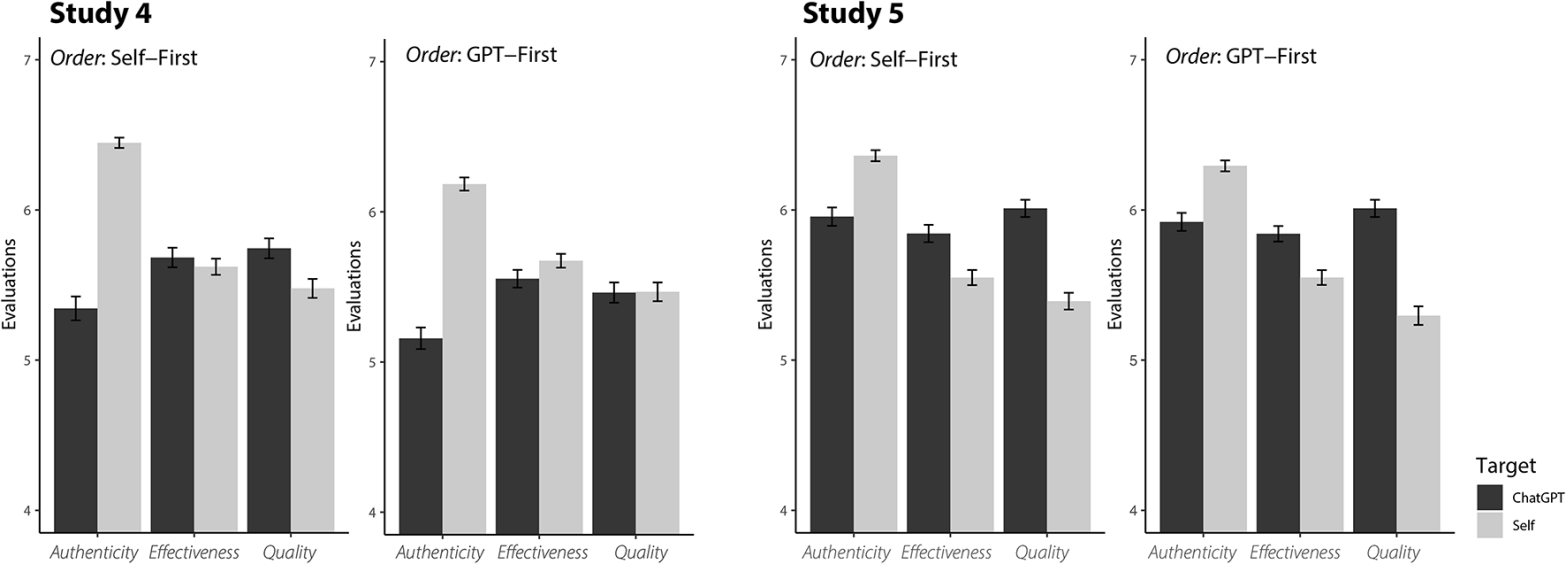
Advice Evaluations for ChatGPT- and Self-Generated Advice by Order. The figure displays the evaluation of the advice by participants for Study 4 (left) and Study 5 (right). These evaluations are split between those that generated and evaluated their advice prior to evaluating ChatGPT advice (right) and vice versa (left). The color represents the target of the ratings, with self-evaluations in gray, lighter bars, and ChatGPT ratings in black, darker bars. The height of the bars represents the mean, and the error bars represent the standard errors.

Crucially, we found that order mattered in two distinct ways. First, people rated ChatGPT-generated advice as higher quality if they tried to generate their own advice first (Cohen’s d = 0.22), suggesting that the effort in self-generating advice remediated the AI bias. Second, people rated their own advice as significantly *less* authentic if they first evaluated AI-generated advice (Cohen’s d = 0.35; see Fig. 3), suggesting that they engaged in social comparison with the AI-generated advice that decreased their evaluations of their own authenticity (see Table 4 for within-person comparisons; see Table 5 for between-person comparisons).

**Table 4:** Within-participant comparisons for advice evaluations (Studies 4-5). Table 4 presents the mean evaluations for self (vs. AI) generated advice in Study 4 (top) and Study 5 (bottom). The columns represent the mean evaluations for ChatGPT and human-generated advice (“M”) and their corresponding standard deviations (“SD”). The right-hand side shaded portion of the table presents the results of within-participant *t-*tests with the mean difference between the variables, with associated *t*-statistic, *p-*value, 95% confidence interval (“Lower CI” and “Upper CI”), and Cohen’s d effect sizes. Recall that in Study 4, participants were aware they were evaluating ChatGPT-generated advice (and self-generated advice). In Study 5, they were told they were evaluating human-generated advice (and self-generated advice).

Variable	TARGET				STUDY 4					
	ChatGPT		Self							
	M	SD	M	SD	Mean diff.	t	*p*-value	Cohen’s d	Lower CI	Upper CI
Effectiveness	5.62	1.19	5.65	0.95	−0.03	−0.49	.624	−0.03	-0.15	0.09
Quality	5.60	1.28	5.47	1.20	0.13	1.70	.091	0.09	-0.02	0.28
Authenticity	5.25	1.44	6.31	0.76	−1.06	−13.66	< .001	−0.72	−1.22	−0.91

**Variable**	**TARGET**				**STUDY 5**					
	**ChatGPT**		**Self**							
	**M**	**SD**	**M**	**SD**	***Mean diff.***	***t***	***p-value***	**Cohen’s d**	**Lower CI**	**Upper CI**
Effectiveness	5.84	1.05	5.55	0.96	0.29	4.86	< .001	0.25	0.17	0.41
Quality	6.01	1.09	5.34	1.12	0.67	9.20	< .001	0.47	0.52	0.81
Authenticity	5.94	1.15	6.33	0.70	−0.39	−6.32	< .001	−0.32	−0.51	−0.27

**Table 5:** Between-participant comparisons for advice evaluations (Study 4). The columns represent the mean evaluations for ChatGPT and human-generated advice (“M”) and their corresponding standard deviations (“SD”). The right-hand side shaded portion of the table presents the results of within-participant *t-*tests with the mean difference between the variables, with associated *t*-statistic, *p-*value, Cohen’s d effect sizes, and 95% confidence interval (“Lower CI” and “Upper CI”).

ChatGPT-Ratings	ORDER				Mean diff.	t	*p*-value	Cohen’s d	Lower CI	Upper CI
	ChatGPT First		Self First							
Variable	M	SD	M	SD						
Effectiveness	5.55	1.12	5.68	1.24	0.13	−1.04	.300	−0.11	−0.37	0.12
Quality	5.46	1.29	5.74	1.26	0.28	−2.11	.035	−0.22	−0.55	−0.02
Authenticity	5.16	1.37	5.34	1.51	0.19	−1.23	.218	−0.13	−0.48	0.11

***Self-Ratings***	**ORDER**				***Mean diff.***	***t***	***p-value***	**Cohen’s d**	**Lower CI**	**Upper CI**
	**ChatGPT First**		**Self** **First**							
**Variable**	**M**	**SD**	**M**	**SD**						
Effectiveness	5.67	0.88	5.62	1.03	−0.05	0.51	.607	0.05	−0.15	0.25
Quality	5.47	1.19	5.48	1.20	0.01	−0.08	.934	−0.01	−0.26	0.24
Authenticity	6.18	0.83	6.45	0.65	0.26	−3.36	.001	−0.35	−0.42	−0.11

In Study 5, we utilized the same paradigm as Study 4. However, we told participants the ChatGPT-generated advice was generated by another human participant. As in Study 2, we again found that the ChatGPT advice was rated more positively. However, this effect was only observed when participants were not told the advice was generated by ChatGPT—participants viewed the ChatGPT-generated advice as higher quality (Cohen’s d = 0.25) and more effective than their own advice (Cohen’s d = 0.47; see Table 4). In line with Study 4, they continued to rate their own advice as significantly more authentic than the ChatGPT-generated advice overall. However, the effect size was significantly smaller (Cohen’s d = 0.32, 95% CI [0.22, 0.43]) than in Study 4 when they were knowingly evaluating ChatGPT (Cohen’s d = 0.72, 95% CI [0.60, 0.83]). There were no significant effects of order when participants were not aware that the advice was generated by an LLM (*p*’s > 0.346).

## Discussion

Artificial intelligence in tools like large language models has begun transforming many aspects of personal and professional life. Given the widespread adoption of these tools, understanding their impact is practically important for users, managers, and policymakers. Although people reported being averse to receiving advice from an AI (Study 1), they evaluate AI-generated advice as higher quality, more effective, and more authentic than human-generated advice (Study 2), a boost that is reduced when participants are aware the advice was generated by ChatGPT (Study 3). In addition, we demonstrate that these results are contingent upon the order in which they were exposed to the advice, and whether they are aware that the advice is generated by ChatGPT (vs. another person; Studies 4–5). While prior work has observed AI-aversion effects, we extend these from numerical outputs to conversational, text-style output in the context of personal advice. In addition, we explore appraisal mechanisms (evaluations of quality, effectiveness, and authenticity) that may lead people to discount this advice. Finally, we document an impact on self-evaluations, specifically self-rated authenticity, that occurs after interacting with ChatGPT-generated advice.

A me-to-machine comparison has implications for social science theories and the future inter-system coordination between people and artificial intelligence. While our findings are focused on the personal advice domain, they collectively underscore the nuanced dynamics at play in the acceptance and evaluation of AI-generated content. The findings regarding the timing of integration with AI-generated content have implications for personal and professional use of these tools.

There are important limitations of the current work worth noting. First, we primarily focus on one large language model—ChatGPT. To generalize beyond this one tool, we replicated Study 2 twice, using two additional LLMs: Claude by Anthropic (Supplemental Study A) and Gemini by Google (Supplemental Study B). We found some variation in the performance of the models in generating advice. However, all models outperformed human-generated advice, being rated as higher quality, more effective, and more authentic (see Supplemental Information for detailed results).

An additional limitation of our studies is that the word “authentic” was in the dating and relationships advice prompt. While this is consistent across all conditions, it may have focused participants’ attention on authenticity ratings. In addition, dating and relationships are a unique context that may be more or less relevant to our participants or their experiences which can impact the advice they generate. With this in mind, we conducted a conceptual replication and extension of Study 4 in Supplemental Study C using a new advice topic—personal development, the second most human-preferred category from Study 1. Notably, our prompt did not contain the word “authentic.” We replicated the key effects of Study 4 with this new prompt and new context (see Supplemental Information for more details).

Further, we measure advice evaluation and intentions to recommend this advice, which is not the same as measuring actual advice uptake. On the one hand, AI aversion may influence evaluations and uptake in tandem where participants indicate they do not like the advice generated by LLMs, and do not take it. On the other hand, the aversion may be limited to evaluations with participants begrudgingly taking the advice because it is of higher quality (as rated in Study 2), even if they say they would not (Study 3). We did not capture this dynamic in terms of behavior, but we believe that an arena for future research unveils the relationship between the AI’s advice and its enactment.

Finally, the mechanism behind our effects – both why people discount ChatGPT-generated advice and why interacting with this advice impacts one’s own authenticity – is unclear. We speculate that ChatGPT and similar large language models are particularly threatening because they are nonhuman agents generating human-like content—here, text-based personal advice. A nonhuman is a strong and salient outgroup to human participants. If this is the case, threat may explain why participants react strongly to the knowledge that the advice was generated by ChatGPT. This threat may also be the cause of the self-distancing occurring in the self-evaluations. Future research should dive more deeply into the mechanisms behind our observed effects.

Our findings add to the careful consideration of the complexities of engaging with LLMs both in the professional domain as well as the personal. Indeed, people’s interactions with algorithms—and how these interactions shape their self-evaluations—have been at the heart of many recent controversies around AI-generated content on social media. Ostensibly, using tools that can generate seemingly credible and creative content^26,27^ could shape people’s evaluations of their capabilities. This is particularly relevant given potential disparities in workplace AI usage amongst jobs, given that AI will be integrated into some jobs more rapidly than others. Taken together, our findings speak to an increasingly valuable domain to understand, as content generated by LLMs becomes a fixture of modern life.

## Methods

All studies were approved by The University of California, Berkeley’s institutional review board. All methods were performed in accordance with the relevant guidelines and regulations. All participants provided informed consent and were compensated for their participation.

### Study 1

**Preregistration**. Data collection, measurement, and analysis follow our preregistration plan can be found here: https://aspredicted.org/JS4_B7W.

**Participants.** As preregistered, we recruited 300 total participants for this study using Prolific Academic. In total, we received 299 responses. After removing participants who did not pass our preregistered quality checks (*n =* 16), our final sample consisted of 283 participants (154 men, 123 women, 3 nonbinary individuals, 3 other-identifying individuals; average age = 34.75 years, SD = 11.24 years; 70.32% White, 16.25% Asian, 11.00% Latino/a, 10.25% Black or African American, 1.06% Other).

A sensitivity power analysis was conducted using G*Power to determine the smallest effect size that we would be able to detect given a power level of 0.80 and an alpha level of 0.05 with 283 participants. The analysis revealed that, under these parameters, the minimum detectable effect size for a one-sample t-test is Cohen’s d = 0.15.

**Procedure.** The goal of this study was to examine preferences for human vs. AI-generated advice across a set of topics. To do this, we gave participants the following information: “People often turn to others for advice. In recent years, large language models such as ChatGPT, Claude, and Gemini offer the chance to ask artificial intelligence (“AI”) for advice. For the following topics, we’d like you to indicate your preference between asking a human for advice vs. asking AI for advice.”

## Measures

**Preference**. As our key dependent variable, participants indicated their preference using a series of slider bars from 0 to 100, with 50 indicating “no preference”. Participants were randomly assigned to see the preference for AI (vs. humans) as 100 or 0 to avoid anchoring effects of the scale. Prior to analysis, we recoded responses to be aligned such that higher values for preferences indicated higher preferences for humans regardless of the scale anchor.

### Study 2

**Preregistration**. Data collection, measurement, and analysis follow our preregistration plan which can be found here: https://aspredicted.org/YY3_RZL.

**Participants.** As preregistered, we recruited 200 total participants for this study using CloudResearch’s approved participant list of Amazon’s Mechanical Turk workers. After removing participants who did not pass our preregistered quality checks (*n =* 32), our final sample consisted of 168 participants (96 women, 69 men, 3 nonbinary individuals; average age = 43.24 years, SD = 12.46 years; 79.76% White, 8.93% Black or African American, 8.33% Asian, 7.74% Hispanic or Latino/a, 2.98% Other).

A sensitivity power analysis was conducted using G*Power to determine the smallest effect size that we would be able to detect given a power level of 0.80 and an alpha level of 0.05 with 168 participants. The analysis revealed that, under these parameters, the minimum detectable effect size for a paired *t-*test is Cohen’s d = 0.22.

**Procedure.** The goal of this study was to examine evaluations of advice, specifically comparing advice generated by ChatGPT, a large-language model developed and maintained by OpenAI, to human-generated advice. To do this, we situated advice in a particular context—dating. Specifically, we asked a separate set of participants and ChatGPT to generate advice for a friend with the following issue: “Say that you had a friend who was asking you about dating. In particular, they were feeling that it’s very difficult to be authentic in a dating context. In a few sentences, what advice would you give that person?”

In total, we had 95 human responses. As an example, one participant wrote:

I would advise my friend to prioritize genuine communication and be true to themselves when dating. Emphasizing shared interests and values fosters authentic connections, while avoiding the pressure to conform to societal expectations. It’s essential to embrace vulnerability and open communication, allowing for a more authentic and meaningful connection with potential partners.

In the survey, participants were randomly presented with one piece of advice from this set. All participants were given the advice as generated by ChatGPT which was:

Authenticity in dating starts with self-awareness and self-acceptance. Take the time to understand your values, interests, and what you’re looking for in a relationship. Be honest with yourself about your strengths and weaknesses. When you have a clear understanding of who you are, you can communicate your true self to potential partners.

They were presented this advice in a counterbalanced order. They completed our key dependent variables after reading each piece of advice.

## Measures

We had three key dependent variables regarding the advice they read. For all items, participants responded to the following prompt, “Please rate the extent to which you agree with the following. This advice is…” for each of the below items. They responded on a 7-point scale where 1 = *Strongly disagree* and 7 = *Strongly agree*.

***Effectiveness***. To measure effectiveness, participants responded to the item “Effective.”

***Quality***. To measure the quality of the advice, participants responded to the item “High Quality.”

***Authenticity***. To measure the perceived authenticity of the advice, participants responded to two items, “Authentic” and “Genuine.” We averaged these items to create our “authenticity” measure.

### Study 3

**Preregistration**. Data collection, measurement, and analysis follow our preregistration plan which can be found here: https://aspredicted.org/ykkr-2tgr.pdf.

**Participants.** As preregistered, we recruited 550 total participants for this study using Prolific Academic. After removing participants who did not pass our preregistered quality checks (*n =* 23), our final sample consisted of 527 participants (306 women, 217 men, 3 nonbinary individuals, 1 genderqueer individual; average age = 38.79 years, SD = 12.98 years; 73.81% White, 12.52% Black or African American, 10.25% Asian, 7.59% Hispanic or Latino/a, 3.42% Other).

A sensitivity power analysis was conducted using G*Power to determine the smallest effect size that we would be able to detect given a power level of 0.80 and an alpha level of 0.05 with 527 participants. The analysis revealed that, under these parameters, the minimum detectable effect size for a between-subjects *t-*test is Cohen’s d = 0.24.

**Procedure.** The goal of this study was to examine biases against ChatGPT-generated advice. To do this, we presented all participants with the same prompt and advice from Study 2. We randomized whether participants were told that ChatGPT (“overt ChatGPT condition”) or another Prolific worker generated this advice (“covert ChatGPT condition”). All participants were given the same advice written by ChatGPT.

## Measures

We had four key dependent variables regarding the advice they read. For all items, participants responded to the following prompt, “Please rate the extent to which you agree with the following. This advice is…” for each of the below items. They responded on a 7-point scale where 1 = *Strongly disagree* and 7 = *Strongly agree*.

***Effectiveness***. To measure effectiveness, participants responded to the item “Effective.”

***Quality***. To measure the quality of the advice, participants responded to the item “High Quality.”

***Authenticity***. To measure the perceived authenticity of the advice, participants responded to two items, “Authentic” and “Genuine.” We averaged these items to create our “authenticity” measure.

***Advice Uptake***. In addition to the quality ratings described above, we also assessed whether participants would recommend someone take this advice. To do so, we utilized a face-valid three-item measure. The items read, “I would recommend this advice to someone in a similar situation,” “I believe others would benefit from following this advice,” and “I would confidently endorse this advice to others” (α = .95).

### Study 4

**Preregistration**. Data collection, measurement, and analysis follow our preregistration plan which can be found here: https://aspredicted.org/6FP_KMW.

**Participants.** As preregistered, we recruited 415 total participants for this study using CloudResearch’s approved participant list of Amazon’s Mechanical Turk workers. To determine our sample size, we conducted a power analysis in G*Power based on an effect size of Cohen’s d = 0.25. This analysis suggested that to achieve 80% power (alpha = 0.05) we should recruit 398 total participants. Given this, we recruited 415 so that we were adequately powered following exclusions.

After removing participants who did not pass our preregistered quality checks (*n =* 51), our final sample consisted of 364 participants (204 women, 154 men, 6 nonbinary individuals; average age = 42.77 years, SD = 13.76 years; 74.73% White, 10.99% Black or African American, 9.89% Asian, 9.07% Hispanic or Latino/a, 3.85% Other).

A sensitivity power analysis was conducted using G*Power to determine the smallest effect size that we would be able to detect given a power level of 0.80 and an alpha level of 0.05 with 364 participants. The analysis revealed that, under these parameters, the minimum detectable effect size for a between-subjects *t-*test is Cohen’s d = 0.29.

**Procedure.** As in Study 2, we were interested in evaluations of ChatGPT-generated advice. Here, we were interested in comparing this advice relative to self-generated advice. All participants evaluated their own advice as well as ChatGPT-generated advice (*within*-subjects comparison). In addition, participants read/wrote advice in a counterbalanced order (*between*-subjects comparison).

For self-generated advice, participants will be asked to generate their own advice to the following prompt, “Say that you had a friend who was asking you about dating. In particular, they were feeling that it’s very difficult to be authentic in a dating context. In a few sentences, what advice would you give that person? Please write at least 3 sentences.”

For ChatGPT advice, they were told, “On the next page, we are going to show you advice written by Chat-GPT -- an artificial intelligence large language model. We will ask you to then evaluate that advice.” The AI advice was as follows: “Authenticity in dating starts with self-awareness and self-acceptance. Take the time to understand your values, interests, and what you’re looking for in a relationship. Be honest with yourself about your strengths and weaknesses. When you have a clear understanding of who you are, you can communicate your true self to potential partners.”

## Measures

We had the same three key dependent variables as Study 2.

### Study 5

**Preregistration**. Data collection, measurement, and analysis follow our preregistration plan which can be found here: https://aspredicted.org/G6G_BNR.

**Participants.** As preregistered, we recruited 415 total participants for this study using CloudResearch’s approved participant list of Amazon’s Mechanical Turk workers. To determine our sample size, we conducted a power analysis in G*Power based on an effect size of Cohen’s d = 0.25. This analysis suggested that to achieve 80% power (alpha = 0.05) we should recruit 398 total participants. Given this, we recruited 415 so that we were adequately powered following exclusions.

In total, we received 413 responses. After removing participants who did not pass our preregistered quality checks (*n =* 33), our final sample consisted of 380 participants (215 women, 160 men, 4 nonbinary individuals, 1 did not disclose; average age = 41.69 years, SD = 12.62 years; 78.68% White, 10.79% Hispanic or Latino/a, 8.68% Black or African American, 6.32% Asian, 2.11% Other).

A sensitivity power analysis was conducted using G*Power to determine the smallest effect size that we would be able to detect given a power level of 0.80 and an alpha level of 0.05 with 380 participants. The analysis revealed that, under these parameters, the minimum detectable effect size for a between-subjects *t-*test is Cohen’s d = 0.28.

**Procedure.** In Study 5, we replicated the procedure of Study 4 with one modification. For the ChatGPT advice, participants were told, “On the next page, we are going to show you advice written by another MTurker. We will ask you to then evaluate that advice.”

## Measures

We had the same three key dependent variables as Study 4.

## Electronic supplementary material

Below is the link to the electronic supplementary material.

Supplementary Material 1

## Data Availability

The data for all studies are available at the following OSF page: https://osf.io/asznb
