# Supplementary material for: Me vs. the machine? Subjective evaluations of human- and AI-generated advice
Source: Sci Rep. 2025 Feb 1;15:3980. doi: 10.1038/s41598-025-86623-6 (PMC11787321; doi:10.1038/s41598-025-86623-6)
Supplement: Supplementary file 1 — Supplementary Material 1 [file 41598_2025_86623_MOESM1_ESM.pdf]

Supplemental Material for

Me vs. the Machine? Subjective evaluations of human- and AI-generated advice

Supplemental Studies A & B

In Supplemental Studies A & B, we replicated Study 2 from the main text with two additional large language models, (A) "Claude" a LLM designed by Anthropic, and (B) "Gemini" a LLM designed by Google. We utilized the same set of advice prompts as Study 2 and the same set of human-generated responses (*N*=95). Our goal was to replicate the higher advice ratings for quality, effectiveness, and authenticity observed in Study 2 for ChatGPT relative to humans for these additional LLMs.

Supp. Study A

Preregistration. Data collection, measurement, and analysis follow our preregistration plan which can be found here: https://aspredicted.org/xf9d-q84z.pdf

Participants. As preregistered, we recruited 200 total participants for this study using CloudResearch's approved participant list of Amazon's Mechanical Turk workers. In total, we received 201 total responses. After removing participants who did not pass our preregistered quality checks (n = 22), our final sample consisted of 179 participants (91 men, 86 women, 1 nonbinary individual, 1 other-identifying individual; average age = 43.75 years, SD = 12.31 years; 78.77% White, 11.73% Black or African American, 7.82% Asian, 5.03% Hispanic or Latino/a, 5.03% Other).

Procedure. The procedure of the study was the same as Study 2. The only difference was that the advice given by ChatGPT was replaced by advice generated by the same prompt by Claude:

> *"Being authentic and true to yourself is so important when dating, even though it can feel challenging. The right person for you will appreciate your genuine self, quirks and all. Focus on being comfortable expressing who you are - your passions, values, and the things that make you unique. That authenticity will shine through and help you find someone who is truly compatible. It may take more time, but it's worth it to find a connection built on mutual understanding and acceptance. The most meaningful relationships come when you can be your unfiltered self."*

Measures

Participants responded to the same dependent variables as Study 2.

Supp. Study B

Preregistration. Data collection, measurement, and analysis follow our preregistration plan which can be found here: https://aspredicted.org/3yt5-y3gf.pdf

Participants. As preregistered, we recruited 200 total participants for this study using CloudResearch's approved participant list of Amazon's Mechanical Turk workers. In total, we received 202 total responses. After removing participants who did not pass our preregistered quality checks (n = 15), our final sample consisted of 187 participants (97 women, 82 men, 2 nonbinary individual, 1 preferred not to say; average age = 44.82 years, SD = 13.72 years; 81.87% White, 9.89% Black or African American, 3.85% Asian, 3.85% Hispanic or Latino/a, 6.04% Other).

Procedure. The procedure of the study was the same as Study 2. The only difference was that the advice given by ChatGPT was replaced by advice generated by the same prompt by Gemini:

“It's completely normal to feel pressure to present a certain way when dating. Remember, authenticity is attractive! Start by focusing on activities you genuinely enjoy and conversations that interest you. Being yourself will naturally draw people who appreciate you for who you are. Don't be afraid to be vulnerable and share your true feelings, even if it feels scary at first.”

Measures

Participants responded to the same dependent variables as Study 2.

Results

Descriptively, of the three LLMs, Claude generated the most authentic and highest quality advice, with Claude and ChatGPT generating similarly effective advice.

Replicating Study 2, both Claude and Gemini generated advice that was rated by participants as significantly higher quality, more effective, and more authentic relative to human-generated advice (see Supp. Figure 1 and Supp. Tables 1-2).

Supplemental Study C

In Supplemental Study C, we conceptually replicated Study 4 from the manuscript in a new domain, personal development. We selected personal development, given that it was the second most human-preferred context from Study 1. In addition, this prompt did not explicitly mention the word "authenticity" in the prompt.

Preregistration. Data collection, measurement, and analysis follow our preregistration plan which can be found here: https://aspredicted.org/h7d7-5q7f.pdf

Participants. As preregistered, we recruited 418 total participants for this study using CloudResearch's approved participant list of Amazon's Mechanical Turk workers. In total, we received 414 total responses. After removing participants who did not pass our preregistered quality checks (*n* = 17), our final sample consisted of 398 participants (202 men, 193 women, 3 nonbinary individuals; average age = 43.37 years, SD = 11.96 years; 78.64% White, 9.30% Black or African American, 8.54% Asian, 6.28% Hispanic or Latino/a, 2.51% Other).

Procedure. We were interested in testing whether the effects observed in Study 3 replicated in a different domain, personal development. All participants evaluated their own advice as well as ChatGPT-generated advice (*within*-subjects comparison). In addition, participants read/wrote advice in a counterbalanced order (*between*-subjects comparison).

We gave participants and ChatGPT the following prompt: "Say that you had a friend who was asking you about personal development. In particular they were finding it very difficult to stick to changes that they want to make in their lives. In a few sentences, what advice would you give that person?"

The ChatGPT advice read: "Making lasting changes is tough, so it's important to start small and be patient with yourself. Focus on just one or two manageable goals at a time, and build consistency by setting routines that fit your current life rather than trying to overhaul everything at once. Tracking progress, celebrating small wins, and forgiving setbacks will help keep you motivated. Finally, stay connected to why the change matters—aligning your goals with your values can make it easier to stay on course."

Measures

Participants responded to the same dependent variables as Study 4.

Results

Replicating Study 4, participants evaluated the AI-generated advice as similar in terms of effectiveness to their advice (Cohen's d's = .11). Replicating Study 4, they rated their own advice as significantly more authentic relative to AI-generated advice (Cohen's d = 0.63). Unlike Study 3, they rated the ChatGPT-generated advice as significantly higher quality than their own advice (Cohen's d = 0.22).

Again, we found that order mattered in two distinct ways. Replicating Study 4, people rated ChatGPT-generated advice as higher quality if they tried to generate their own advice first (Cohen's d = 0.26). Second, people rated their own advice as significantly *less* authentic if they first evaluated AI-generated advice (Cohen's d = 0.21), suggesting that they engaged in social comparison with the AI-generated advice which decreased their evaluations of their advices' authenticity (see Supplemental Table 3 for within-person comparisons; see Table 4 for between-person comparisons).

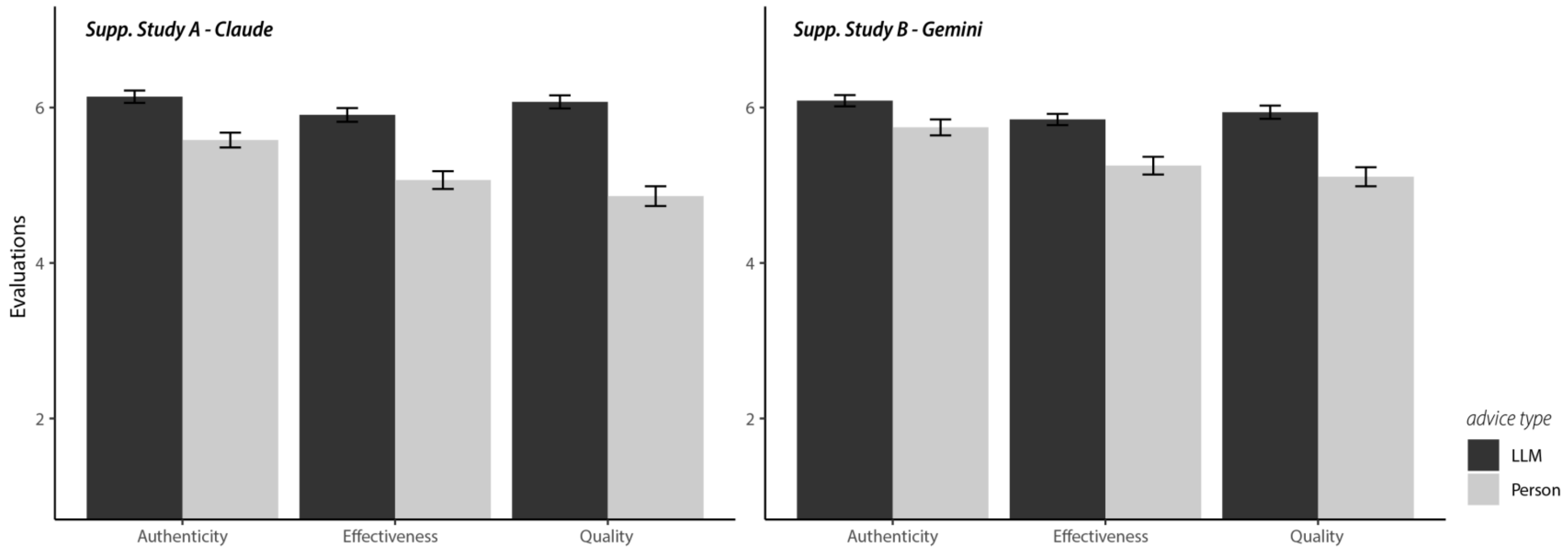

Supp. Fig. 1. Advice Evaluations for Claude-, Gemini- and Human-Generated Advice. The figure displays the evaluation of the advice by participants. The evaluations of Claude (left) and Gemini (right) are presented in the darker bars, with evaluations of human-generated advice in the lighter bars. The bars represent the mean, and the error bars represent the standard errors.

	TARGET									
	Claude		Human							
**Variable**	**M**	***SD***	**M**	***SD***	***Mean diff.***	***t***	***p-value***	**Cohen's d**	**Lower CI**	**Upper CI**
Effectiveness	5.91	1.14	5.07	1.49	0.84	7.16	<0.001	0.54	0.61	1.07
Quality	6.07	1.10	4.86	1.64	1.21	9.23	<0.001	0.69	0.95	1.47
Authenticity	6.14	1.03	5.58	1.24	0.56	5.44	<0.001	0.41	0.36	0.76

Supp. Table 1. Within-Participant Comparisons for Advice Evaluations (Supp. Study A). Supp. Table 1 presents the mean evaluations for human (*vs*. AI) generated advice in Supp. Study A. The columns represent the mean evaluations for Claude and human-generated advice ("M") and their corresponding standard deviations ("SD"). The right-hand side shaded portion of the table presents the results of within-participant *t*-tests with the mean difference between the variables, with associated *t*-statistic, *p*-value, 95% Confidence Interval ("Lower CI" and "Upper CI"), and Cohen's d effect sizes.

	TARGET									
	Gemini		Human							
**Variable**	**M**	***SD***	**M**	***SD***	***Mean diff.***	***t***	***p-value***	**Cohen's d**	**Lower CI**	**Upper CI**
Effectiveness	5.85	0.94	5.25	1.47	0.59	5.33	<0.001	0.40	0.37	0.81
Quality	5.94	1.10	5.11	1.59	0.83	6.71	<0.001	0.50	0.59	1.07
Authenticity	6.09	0.94	5.74	1.32	0.34	3.22	0.002	0.24	0.13	0.55

Supp. Table 2. Within-Participant Comparisons for Advice Evaluations (Supp. Study B). Supp. Table 2 presents the mean evaluations for human (*vs*. AI) generated advice in Supp. Study A. The columns represent the mean evaluations for Gemini and human-generated advice ("M") and their corresponding standard deviations ("SD"). The right-hand side shaded portion of the table presents the results of within-participant *t*-tests with the mean difference between the variables, with associated *t*-statistic, *p*-value, 95% Confidence Interval ("Lower CI" and "Upper CI"), and Cohen's d effect sizes.

	TARGET									
	ChatGPT		Self							
**Variable**	**M**	**SD**	**M**	**SD**	***Mean diff.***	***t***	***p-value***	**Cohen's d**	**Lower CI**	**Upper CI**
Effectiveness	5.62	1.19	5.65	0.95	-0.03	-0.49	0.624	-0.03	0.17	0.09
Quality	5.60	1.28	5.47	1.20	0.13	1.70	0.091	0.09	0.52	0.28
Authenticity	5.25	1.44	6.31	0.76	-1.06	-13.66	<0.001	-0.72	-0.51	-0.91

Table 3. Within-Participant Comparisons for Advice Evaluations (Supp. Study C). Table 3 presents the mean evaluations for self (*vs*. AI) generated advice in Supplemental Study C. The columns represent the mean evaluations for ChatGPT and human-generated advice ("M") and their corresponding standard deviations ("SD"). The right-hand side shaded portion of the table presents the results of within-participant *t*-tests with the mean difference between the variables, with associated *t*-statistic, *p*-value, 95% Confidence Interval ("Lower CI" and "Upper CI"), and Cohen's d effect sizes.

	ORDER									
ChatGPT-Ratings	**ChatGPT First**		**Self First**							
**Variable**	**M**	**SD**	**M**	**SD**	***Mean diff.***	***t***	***p-value***	**Cohen's d**	**Lower CI**	**Upper CI**
Effectiveness	5.68	1.24	5.55	1.12	0.13	-1.04	0.300	-0.11	-0.37	0.12
Quality	5.74	1.26	5.46	1.29	0.28	-2.11	0.035	-0.22	-0.55	-0.02
Authenticity	5.34	1.51	5.16	1.37	0.19	-1.23	0.218	-0.13	-0.48	0.11
	**ORDER**									
Self-Ratings	**ChatGPT First**		**Self First**							
**Variable**	**M**	**SD**	**M**	**SD**	***Mean diff.***	***t***	***p-value***	**Cohen's d**	**Lower CI**	**Upper CI**
Effectiveness	5.62	1.03	5.67	0.99	-0.05	0.51	0.607	0.05	-0.15	0.25
Quality	5.48	1.20	5.47	1.19	0.01	-0.08	0.934	-0.01	-0.26	0.24
Authenticity	6.45	0.65	6.18	0.83	0.26	-3.36	0.001	-0.35	-0.42	-0.11

Table 4. Between-Participant Comparisons for Advice Evaluations (Supp. Study C)*.* The columns represent the mean evaluations for ChatGPT and human-generated advice ("M") and their corresponding standard deviations ("SD"). The right-hand side shaded portion of the table presents the results of within-participant *t*-tests with the mean difference between the variables, with associated *t*-statistic, *p*-value, Cohen's d effect sizes, and 95% Confidence Interval ("Lower CI" and "Upper CI").
